# Using a complex adaptive system lens to understand family caregiving experiences navigating the stroke rehabilitation system

**DOI:** 10.1186/s12913-016-1795-6

**Published:** 2016-10-01

**Authors:** Andrea Ghazzawi, Craig Kuziemsky, Tracey O’Sullivan

**Affiliations:** 1 Telfer School of Management, University of Ottawa, 55 Laurier Ave E, Ottawa, ON K1N 6 N5 Canada; 2 Interdisciplinary School of Health Sciences, University of Ottawa, 55 Laurier Ave E, Ottawa, ON K1N 6 N5 Canada; 3 Bruyère Research Institute, 55 Laurier Ave E, Ottawa, ON K1N 6 N5 Canada

**Keywords:** Caregiving, Complex adaptive systems, Navigation, Stroke rehabilitation, Ageing

## Abstract

**Background:**

Family caregivers provide the stroke survivor with social support and continuity during the transition home from a rehabilitation facility. In this exploratory study we examined family caregivers’ perceptions and experiences navigating the stroke rehabilitation system. The theories of continuity of care and complex adaptive systems were integrated to examine the transition from a stroke rehabilitation facility to the patient’s home. This study provides an understanding of the interacting complexities at the macro and micro levels.

**Methods:**

A convenient sample of family caregivers (*n* = 14) who provide care for a stroke survivor were recruited 4–12 weeks following the patient’s discharge from a stroke rehabilitation facility in Ontario, Canada. Interviews were conducted with family caregivers to examine their perceptions and experiences navigating the stroke rehabilitation system. Directed and inductive content analysis and the theory of Complex Adaptive Systems were used to interpret the perceptions of family caregivers.

**Results:**

Health system policies and procedures at the macro-level determined the types and timing of information being provided to caregivers, and impacted continuity of care and access to supports and services at the micro-level. Supports and services in the community, such as outpatient physiotherapy services, were limited or did not meet the specific needs of the stroke survivors or family caregivers.

**Conclusion:**

Relationships with health providers, informational support, and continuity in case management all influence the family caregiving experience and ultimately the quality of care for the stroke survivor, during the transition home from a rehabilitation facility.

**Electronic supplementary material:**

The online version of this article (doi:10.1186/s12913-016-1795-6) contains supplementary material, which is available to authorized users.

## Background

With increasing life expectancy, an ageing population, and rising rates of obesity and diabetes, the incidence of stroke is projected to rise [1]. Fifteen million people worldwide experience a stroke each year and approximately one-third die; with another one-third are left disabled from the stroke [2]. In Canada, stroke is the third leading cause of death, and the leading cause of disability [1]. As a result, more Canadians who have had a stroke are living with its effects [1]. The type and extent of disability varies, but may include difficulty communicating, performing activities of daily living and personal care, as well as depression [3]. Stroke not only affects the individual, but also has significant impacts on the family, and is therefore considered a ‘family disease’ [4].

Family caregivers play an instrumental role throughout the stroke trajectory by providing a consistent source of support for the stroke survivor across health care settings [4, 5]. We define the caregiving dyad as the care recipient and family caregiver, who is often a family member or spouse providing the care recipient with social support and care. However, with the increasing complexity of the stroke rehabilitation system, the caregiver-patient dyad experience a greater number of transitions across the continuum of care from emergency to acute care, rehabilitation and community care, and sometimes back to the acute or rehabilitation facility [5].

Achieving continuity of care is challenging in the stroke rehabilitation system and the family caregiver is often the only aspect that remains consistent as a patient transitions across care settings [6]. As such, the family caregiver is often responsible for scheduling medical appointments, coordinating community services, and providing the stroke survivor with instrumental and emotional support [5]. One significant role played by the family caregiver is the system navigator that locates, evaluates, and integrates knowledge and information [7]. However, coordinating services can be a complex process as there is often little or no continuity in homecare and/or rehabilitation services, resulting in feelings of abandonment and isolation [8, 9]. This impacts the stroke survivor’s momentum toward recovery, as rehabilitation is a not an event but rather a journey in which the stroke survivor transitions away from disability [8].

Multiple attributes in the stroke rehabilitation system impact the care provided to patients and the family caregiver’s ability to fulfill their role as system navigator and support provider. The number of attributes and degree of interaction between them must be considered in designing interventions to support caregivers and stroke survivors. White et al. [10] conducted a systematic review examining interventions such as education, information, counseling and training for family caregivers of stroke survivors. They concluded that multi-faceted interventions were more likely to show a positive effect, while singular interventions showed positive impacts that diminished over time [10]. The delivery of sustainable supports and services for family caregivers and stroke survivors in the community setting continues to be a challenge [11].

### Continuity of care

Continuity of care has been identified as an essential element in the provision of quality health services [12–14]. However, with an ageing population and the growing complexity of the health care system, health professionals, patients and policy-makers are increasingly concerned about fragmentation of care [1]. Continuity of care is important during the transition between health care settings, with good continuity providing benefits including increased patient satisfaction [13–15], fewer hospitalizations and fewer emergency department visits [12, 16, 17]. However, family caregivers of stroke survivors report lack of support from health professionals, insufficient information and difficulty accessing resources as they transition across the continuum of stroke care, all of which impact continuity of care [18–20].

Research into continuity of care has been identified as a priority [21] and it has been examined in a number of areas, such as stroke care, primary care and mental health services [15, 22–25]. While conceptual in nature, continuity of care has also been studied in combination with other models and measures, such as the Bice-Boxerman measure [14, 18, 26].

Haggerty, Reid, Freeman, Starfield, Adair, & McKendry [27] identified three elements of continuity in the provision of health care services: 1) informational, 2) relational and 3) management continuity. Informational continuity is defined as the information that links both providers and health care events and can include information on patient medical conditions, values, and/or preferences [27]. Relational continuity connects past and current health care with future care, ensuring a continuous relationship between health care providers and their patients across care settings [27]. Management continuity, focuses on the overall vision of treating a specific health problem or chronic condition. Management continuity examines the provision and organization of health care services over time in order to enhance quality of life [22, 27]. Further research is needed to examine the complexities of how continuity of care impacts the caregiver-patient dyad experiences navigating the stroke system.

### Complex adaptive systems (CAS)

Systems thinking, particularly about complexity and complex adaptive systems became more widely known in the 1980’s with the creation of the multi-disciplinary think tank, called the Santa Fe Institute [28]. Complexity is defined as the inter- and intra-relatedness of the components of a system [29]. Tenets common to CAS include, feedback, emergent behaviours, non-linear processes, co-evolution, requisite variety, and simple rules [30–32]. As a system becomes more complex, the number of components and interactions between each component increases within a system and between a system and its environment [29]. CAS is a problem solving approach that acknowledges the complexity of organizations and networks, and the relationships within and between system components [29]. Ellis & Herbert [31] also provide a means for examining the complexity and dynamic nature of organizations and networks at the system levels (e.g. macro, meso, micro).

CAS is a lens for understanding the health care system as it provides a way to understand non-linear and dynamic properties, as well as the manner in which complexity increases over time [33–35]. Research on health systems and management has adopted CAS to better understand multilevel organizational behaviour within the healthcare system [29, 30, 35–38]. Kannampallil et al. [29] provides a thorough explanation of how the CAS lens can be used to better understand the relationships between system components in health care, such as emergency department transfers to the intensive care unit, and workflow modeling and critical care.

Maintaining continuity of care across multiple settings and providers is challenging due to the increasing number of components and the high degree of interrelatedness between the system’s components [29]. More specifically, continuity of care in the stroke rehabilitation system has been impacted by the increased complexity of the health care system resulting in a greater number of transitions between health care settings [39]. While research has examined family caregivers’ experiences caregiving for a stroke survivor as they transition across the stroke trajectory [5, 40, 41], they have not been studied through a CAS lens. This perspective provides an opportunity to more fully understand the dynamic complexity of the stroke rehabilitation system and how to better integrate inpatient and community settings to support continuity of care.

In this exploratory study we address the above need by studying family caregivers’ perceptions and experiences navigating the stroke rehabilitation system. The theories of continuity care and CAS were integrated to examine the transition from a stroke rehabilitation facility to the patient’s home. We provide an understanding of the interacting complexities at the macro and micro levels, specifically the inter- and intra-relatedness between the system components that impact continuity of care and the family caregiving experience.

## Methods

This exploratory study used a qualitative methodology to examine family caregivers’ perceptions and experiences navigating the stroke rehabilitation system.

### Data sources

Purposeful sampling was used to recruit primary family caregivers of stroke survivors who were receiving care as outpatients at the rehabilitation center and recovering at home at the time of data collection. The family caregivers were approached by a social worker at the rehabilitation facility. If they agreed to receive additional information about the study, the family caregivers were contacted within 48 h of being approached. Twenty participants were contacted and 14 agreed to participate in the study during a recruitment period of 6 months (from March to September); 6 caregivers declined participation due to caregiving demands. To be eligible, at the time of participation, the stroke survivor must have been living at home for 4 − 12 weeks following post-discharge from a rehabilitation facility. Family caregivers of stroke survivors who were no longer receiving inpatient rehabilitation but were residing in the community were also eligible to participate in the study.

Ethics approval was obtained from both the rehabilitation facility and the University of Ottawa prior to commencing data collection. A completed consent form was received from each participant prior to conducting the semi-structured interview and was reviewed at the start of the interview. To accommodate participant needs and preferences, interviews were conducted in person, or by telephone using a semi-structured interview guide. Stroke survivors were not present during the interviews. Prior to the interview, participants were provided with the consent form and a summary sheet with a definition of continuity of care. Five questions were asked; some questions had multiple parts and were open ended, to gain insight on the experiences and barriers of providing care. Interviews were 30−45 min in length. The interview notes taken during and after each interviews as well as the electronic recordings were transcribed verbatim.

### Data analysis

The coding tree (see Additional file 1) was developed by all 3 authors. Components from the continuity of care theory were included as direct codes and the remainder of the tree was developed inductively. The first and third authors independently reviewed 2 transcripts to identify potential labels for the coding tree. These labels were refined into a preliminary coding tree, which was iteratively discussed by all 3 authors until consensus was reached. All the transcripts were then coded by the first author using NVivo 9 qualitative software. Coding reports were reviewed by the other authors with regular meetings held to discuss data coding and to make decisions on codes that were not clear. Directed and inductive content analysis was used to analyze the research data. Preliminary themes were identified by the first author and discussed with the other authors throughout the analysis phase. Saturation was reached after 14 interviews, when no new themes emerged. The complexity lens was then applied in order to identify how the tenets of complex adaptive systems related to the emergent themes from the study. A summary of the transcripts and/or findings was not sent to the participants for verification.

For the purpose of this study, the micro-level focused on the caregiver-patient dyad as the unit of analysis, whereas the macro-level examined policies, procedures, information, services etc. that influence the micro level dyads. This provided an understanding of how complexity at the macro and micro levels impacts the family caregiver’s ability to navigate the system during the transition from a rehabilitation facility to home.

## Results

In total, *n* = 14 family caregivers completed interviews for the study. Eight participants were female and 6 male. The median age was 63 years. Six family caregivers were employed full-time and 9 stroke survivors experienced communication difficulties and/or cognitive impairment due to stroke. Nine family caregivers did not report any health conditions; however, 5 reported suffering from chronic health conditions such as high blood pressure, diabetes, and arthritis.

Complexity exists at both macro and micro levels and the 2 levels interact to impact continuity, and ultimately quality of care for the stroke survivor. Our results are presented in 2 main parts. First, we use the tenets of CAS to articulate the macro level issues [30–32]. Second, we describe the interaction of these issues at the micro level from the perspective of roles, supports and services, and information.

**Fig. 1:**
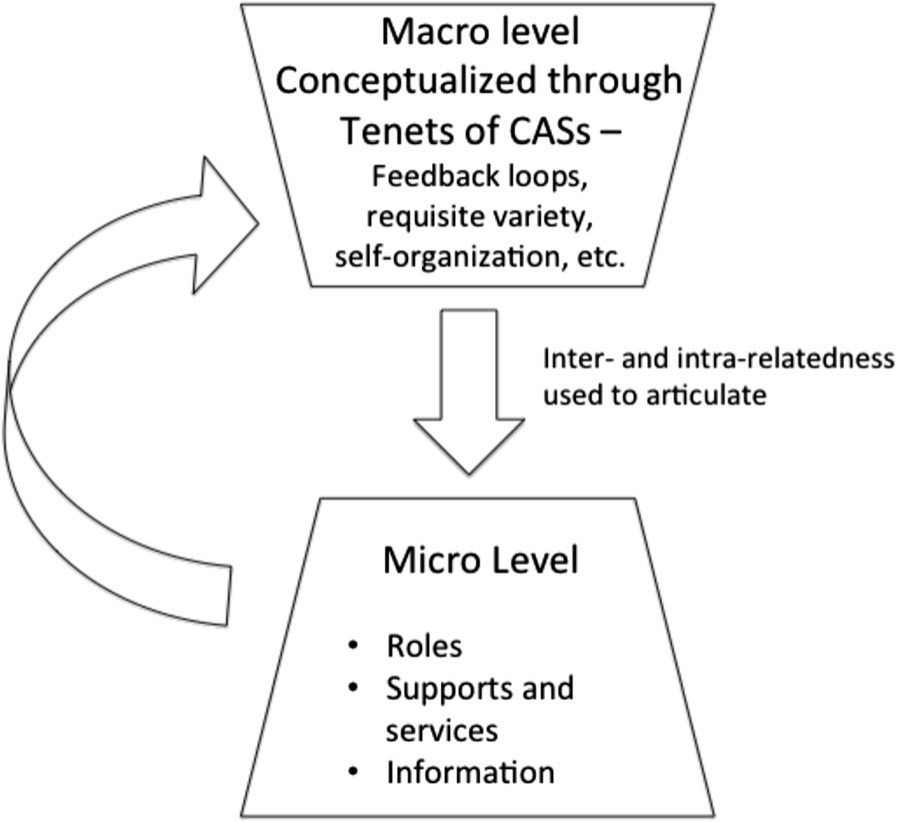
Interaction between the macro and micro-levels. Illustrates our analysis of the family caregiving experience in the stroke rehabilitation system using complex adaptive systems. The tenets of CAS were used to articulate the macro level issues. We then described the interaction of these issues at the micro level from the perspective of roles, supports and services, and information

**Fig. 2:**
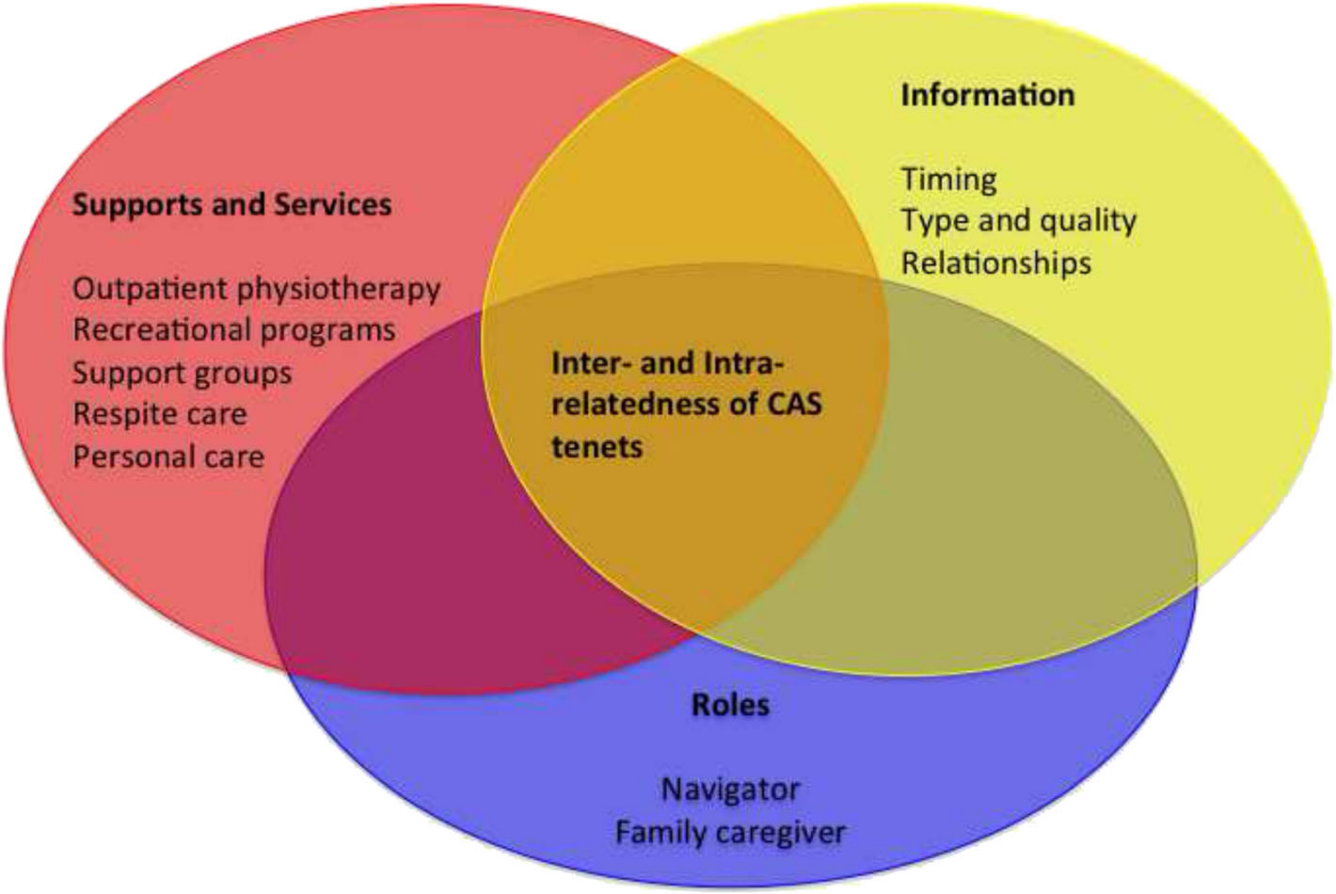
Micro-level overarching categories. Illustrates the three overarching categories, the themes that emerged for each category, and the inter- and intra-relatedness between the different aspects of CAS and the micro categories

Figure 1 illustrates the high-level trickle-down effect from the macro-level to the micro-level. To study the relationship between the micro and macro levels we studied family caregiving at the micro level by analyzing the inter- and intra-relatedness of the CAS tenets. From that analysis we identified roles, supports and services, and information as the 3 overarching categories that impact family caregivers at the micro level. These categories are consistent with other literature on care provision in the complex health networks e.g [33–35]. Figure 2 expands on Fig. 1 by presenting the 3 overarching micro categories, the themes that emerged for each category, and the inter- and intra-relatedness between the CAS tenets and the three overarching micro categories. We will now discuss the macro and micro-level analysis of the family caregiving experience.

### A macro-level analysis of the family caregiving experience

Table 1 maps the family caregivers’ experiences navigating the stroke rehabilitation system at the macro-level during the transition home from a rehabilitation facility, using the tenets of complex adaptive systems. This table also illustrates various examples of how macro level complexity impacted the family caregiving experience at the micro-level illustrated in Fig. 1. Simple rules at the macro-level, such as facility policies and procedures are what structure and regulate the provision of care. However, non-linear processes result in complexity at the macro level due to altered demand for services, which then trickles down to the micro-level. For example, early discharge from the rehabilitation facility will caused increased demand and therefore impact access to supports and services in the community, such as outpatient physiotherapy. Continuity of care may also be interrupted by co-evolution or emergent behavior due to the system’s inability to adapt to variations in the family caregiving experience. While formal processes may exist at the macro level, if they are not timed well or a good fit with individual contexts at the micro-level, then people self-organize to find solutions to problems. Self-organization or emerging behavior was more prevalent in situations where caregivers had extensive social supports and/or previous experiences in the healthcare system. The information and knowledge was then fed back into the system from the caregivers through feedback loops, contributing to management of care.

**Table 1:** A macro-level analysis of the family caregiving experience using complex adaptive systems

Tenets of complex adaptive systems	Application to the caregiving experience
Non-linear processes	Changes to one area impact other areas in direct and indirect patterns. For example, early discharge from the stroke rehabilitation facility impacts the outpatient physiotherapy wait-list, and access to those services.
Self-organization	The actor’s ability and tendency to adapt to the complex nature of the system. For example, professionals bending protocols or bringing in non-traditional resources.
Emergent behaviour	Behaviours that emerge as a result of the dynamic and complex nature of the system. For example the way an actor adapts to a specific event such missing information.
Feedback loops	Transfer of knowledge or information in response to an experience. For example information and knowledge was fed back into the system from the caregivers, contributing to management of care.
Co-evolution	The role of each actor changes according to the unique needs and attributes of the case. For example, the development of increased awareness of procedures and protocols at the facility and during transitions.
Requisite variety	The unique characteristics of the case, or actors involved, influence the changing context in continuity of care. For example, unique attributes such as past experiences, beliefs, medical knowledge, and social networks, influenced case management.
Connectivity	The relationships between actors in the system. For example, the relationships between actors involved in the case impact communication and information exchange.
Simple rules Non-discrete boundaries	The discovery of rules and boundaries within the system. Examples of rules and boundaries include, facility policies, procedures, and protocols, such as discharge procedures.

### A micro-level analysis of the family caregiving experience and interacting complexities

This section will discuss the interacting complexities, more specifically the inter- and intra-relatedness of the CAS tenets that impact family caregivers during the transition home from a rehabilitation facility. Roles, supports and services, and information emerged as the three overarching categories that impact the family caregiving experience at the micro level. Roles are the functions assumed by the family caregiver, such as system navigator or family care provider, during the transition home from a rehabilitation facility. Supports and services refer to the provision of care or assistance provided to the family caregiver or stroke survivor, such as outpatient physiotherapy, support groups and homecare. Information refers to written or oral facts, regarding for example supports and services in the community or the stroke survivors’ care plan, which are provided to the family caregiver and stroke survivor.

### Roles

The family caregiver is a constant source of support for the stroke survivor throughout the stroke trajectory, but the role becomes more complex after the transition home. Several family caregivers described being a “gatekeeper” between the stroke survivor and friends/other family members, while the stroke survivor was at the facility. *“He had probably 20 friends who emailed me regularly asking for his status.” (P7)* At home, some family caregivers felt the need to monitor the stroke survivor, and that they are *“really constantly on call for everything.” (P14).* However, requisite variety, specifically the unique attributes of each case such as the stroke survivors’ mental, physical (functional), and emotional needs, or the family caregiver’s own health and well-being, impacted the caregiving role. For example, family caregivers in the study who had their own serious health conditions, found providing care for the stroke survivor more complex, and had to reach out for supports and services in the community for assistance. However, availability of services is sometimes an issue, further impacting the degree of complexity experienced by the family caregiver at the micro-level. A consequence was that some family caregivers reached out again to their social support networks for assistance to ease the caregiving role and help decrease complexity at the micro-level.

An emergent role for all of the caregivers was that of a ‘system navigator’. This role emerged because of gaps in continuity of care – which varied in nature according to individual patient contexts. For example, care facilities had formal processes, such as facility procedures and protocols that govern when information is provided and how tasks are scheduled and done (refer to Table 1). One caregiver described how while they received a generic book of available services it was up to them to find out the specifics of what services were offered and their availability. *“You can get a book that lists 10 different agencies. You don’t know who does what for who, so you call them and you are on hold, and you are worried.” (P2).* Family caregivers also often had emerging navigator roles to advocate for the stroke survivor at the rehabilitation facility, as well as to fulfill administrative roles and responsibilities, such as scheduling medical appointments, or filing and organizing paperwork.

*“So yeah so you have to keep—you have to be on top of things and say “Well look is that still on or is that still off?” and I had to cancel one appointment and so— you have to keep tabs to make sure that you know everything’s going according to plan because sometimes someone might forget to call so and so…” (P3)*

An emerging behavior was that several family caregivers in the study used their social support networks as a source of navigational support and drew from their past experiences, and/or medical background, in order to help manage navigational complexity. Finally, as part of the navigator role, the family caregivers were an informational link for the health professionals involved in the stroke survivor’s care. Caregivers described pushing and pulling information about the stroke survivor’s health status or care, and/or services and supports, from the health professionals, as part of maintaining continuity. However, for some the information broker role eventually became burdensome and some caregivers lost their desire to continue pulling and pushing information. *“So I kind of lost my desire to ask more questions at that point, I felt tired of pulling the information out versus the information being offered.” (P7).*

### Supports and services

The degree of complexity at the macro-level, such as the availability of financial resources or human resources in the system, greatly impacted family caregivers’ abilities to access supports and services in the community. Requisite variety in each case, such as proximity to services, weather, or financial situation impacted access to services, added micro-level complexity and impacted continuity of care (refer to Table 1). Many family caregivers discussed waiting for extended periods of time for services (e.g. outpatient physiotherapy). Due to the increased wait-times, many families were required to decide whether or not to pay for private services, especially for outpatient physiotherapy, given its importance for continuity of care in stroke rehabilitation. However, in some cases the families decided the cost outweighed the benefit.

*“I could have gotten a physio to come in once a week, but you are talking, I think $100 a visit, and I think my plan only pays $400 so I didn’t see the benefit of that […] I could have done it for 4 visits I guess, but what is 4 visits going to do when we knew we were going to get to out patient [physio]… you know what I mean, I weighed the two.” (P2)*

Location was another complexity factor. Family caregivers in the study who lived outside the city limits found accessing services more complex as the services may not be available in rural areas. As described by one caregiver, *“Well yes, I’ve gone through them but the services that are listed are mainly for people residing in the city. [in rural areas] It was not helpful as far as the services are concerned.” (P12).* A further challenge was that while accessing the services in good weather conditions was an option, family caregivers often were often hesitant to drive into the city in winter conditions to access services.

### Information

The coordination and communication of information were important during the transition home from a stroke rehabilitation facility. Simple rules and boundaries, timing of information and type and quality of information, were identified as factors influencing information exchange during this transition (refer to Table 1). Legal boundaries were one type of rule that governed information exchange between health professionals, the stroke survivor and the family caregiver. At times these simple rules prevented the ability to obtain necessary information. .

*“Trying to get him into the right areas - it’s been hard, but the hardest thing I find is getting information about his medical conditions, because I’m not power of attorney, so there is that confidentiality issue and just trying to navigate through getting his prescriptions set up for him and I can’t get any information” (P1)*

Another significant challenge to information continuity was the timing of information that was provided to family caregivers. Formal processes such as facility procedures and protocols, typically governed when information is provided to the family caregiver, which was usually at discharge (refer to Table 1). However, continuity of care is a temporal activity and its context is defined by the requisite variety of each unique situation. The information provided at discharge is often not what is needed at the specific time of need, which adversely impacts the family caregiver’s ability to self-organize and adapt in their role as system navigator.

The relationships or connectivity between the family caregiver and health professional influenced complexity at the micro-level, as it was a barrier or facilitator to communication depending on the context. Simple rules and boundaries in the system also governed the formality of these relationships (refer to Table 1). Many family caregivers felt comfortable and found the interaction with professionals positive. However, family caregivers who experienced poor relational continuity with the health professionals often lost trust and their desire to maintain the relationship and receive information or guidance from the health professional. As described by one family caregiver, *“And the hardest part for me was getting information, I felt that I was inconveniencing [the health care professional] … [it] was extremely negative.” (P1).* Instead the caregiver turned to other information sources such as the Internet, or other people, to assist with navigational complexity.

## Discussion

Family caregivers play an instrumental role throughout the stroke trajectory from the onset of stroke to recovery. The caregiving role during the transition from a rehabilitation facility to home is particularly important as the care shifts from the health professionals to the family caregiver [5]. The findings from this study echo existing research in that discharge from a rehabilitation facility to home is a critical time of adjustment and requires learning and adaptation for the family caregiver and stroke survivor [5, 42–44]. This study contributes to the wider body of literature by using complex adaptive systems (CAS), a non-linear perspective, to provide insight on the caregiving process, specifically on the micro and macro interactions that occur during the transition home from a rehabilitation facility: a key transition in the caregiving experience. This study also illustrates the inter- and intra-relatedness between information, supports and services, and roles, as well the complexity that exists at the micro level impacting continuity, and ultimately quality of care for the stroke survivor.

A key premise of CAS is the need to view healthcare delivery as a complex rather than a mechanical system [29]. This perspective enables us to use CAS properties (e.g. non-linearity, simple rules) to gain an understanding of the system and to better manage complexity in order to develop policy and other interventions to help shape the behavior of the system and mitigate barriers to care. While continuity of care is essential to support the seamless transition between health care settings, complexities such as finances, proximity to care, and relationships, emerged as barriers impacting the caregiving role.

In our study the non-linear and dynamic nature of the stroke rehabilitation system required family caregivers to adapt their role to include tasks such as system navigator or gatekeeper of information and support and services. Cameron and Gignac [5] illustrate the family caregivers need to adapt in their model depicting the caregivers’ experience and needs over time as the stroke survivor transitions across care settings. The navigator role was made more complex because of the requisite variety of the caregiving experience and varied depending on the health status of the caregiver and stroke survivor and financial considerations of the services. While the costs for required services such as physical therapy are covered in hospital they may be the patient’s responsibility in the community. Without private insurance, patients may not be able to pay for services, which can have an impact on recovery. The finding from this study aligns with Campbell et al. [45] in that finances are a barrier for many Canadians with cardiovascular-related chronic conditions and impact their ability to access health care services in the community. Further, in our study participants from rural locations said that the required services may not even be available. Access to health care services in rural locations remains an ongoing issue for community members requiring health related supports and services [46]. The non-linearity and requisite variety o the above situations brought about emergent behaviors by the caregiver by acting as a navigator in an attempt to procure necessary services.

Our study participants emphasized the importance of informational continuity in managing the stroke rehabilitation system but they also described significant barriers to achieving it. Family caregivers often had to act as a hub of information that involved pushing information to and pulling information from the health professionals about the stroke survivor’s health status or care, and/or services and supports. However, many family caregivers reported receiving insufficient information. The findings from this study mirror existing research in that family caregivers report receiving insufficient information during the transition home from a rehabilitation facility [18, 19]. That issue caused some family caregivers in our study to use their social support networks as an informational resource, whereas others developed research skills to self-organize and utilize the Internet as a source of information. However, the Internet is not always a reliable source of information, particularly in the context of complex and context dependent scenarios such as stroke rehabilitation on the community. This issue highlights the need for community driven solutions that move beyond the current structure and are tailored for each individual patient and family’s needs.

The timing of information was another significant factor impacting informational continuity. Family caregivers described how formalized procedures and protocols in the healthcare system, such as discharge procedures at the facility, determine when information is provided to the caregiver-patient dyad. However, discharge was described by the family caregivers in our study as a time of extensive adjustment. Literature identifies the transition home from a rehabilitation facility as a critical time of adjustment [5, 19, 44]. Receiving high volumes of information at that time resulted in a deluge of information which many caregivers in our study found to be overwhelming, rendering the information useless for many family caregivers and adversely impacting their role as system navigators. Due to the poor timing of information, the family caregivers in our study were often required to self-organize in order to adapt to the overload or gap in information.

Based on the results of this study we offer the implications for practice that could help reshape processes to better support complex activities like stroke rehabilitation, and the family caregiver’s role in it, during the transition home from a rehabilitation facility.

### Tailoring the timing of information

The timing of information is established at the macro-level which influences complexity at the micro-level. By tailoring the timing of information to the needs of the family caregivers, it would ensure that information is not provided too early or too late, but at the time of greatest need. Each caregiver’s unique situation needs to dictate the provision of information. Care continues to transition into the community. The coordination and communication between community organizations and health care facilities could help facilitate improved timing of information. This will better support the family caregiver’s role as system navigator and reduce the deluge of information improving the family caregivers’ perceived experiences navigating the stroke rehabilitation system.

### Streamline the information being provided

Providing family caregivers with information specific to their needs and community contexts could improve the utilization of supports and services in the community, and reduce complexity experienced at the micro-level by the family caregivers. Information delivery needs to be specific to the context of each patient and aligned with the services that are available to a patient as well as the patient’s ability to access services. If necessary services are not accessible, and/or context specific information is not provided, then trust can erode (as discussed in the Information section above), requiring the family caregiver to self-organize in order to find solutions.

### Provide a case manager or system navigator to assist the family caregiver

The introduction of a case manager or system navigator could ease the family caregiver’s role, reduce complexity at the micro-level and improve the provision of care by supporting continuity of care across health care settings. This resource could be in the form of an application for a smart phone or a human resource in the health care system. This resource would be an informational support for family caregivers that would help prepare them for their caregiving role and then inform them of available supports and services that are tailored to the specific context of a patient’s transition from a rehabilitation facility to home.

### Limitations

There are limitations in this study that need to be acknowledged. The first limitation is participant recruitment, which was conducted at one rehabilitation facility, using purposive sampling that limits the generalizability of this study’s research findings. Also, one interview was conducted per participant. Transcripts and findings were not returned to the participants for verification. Most of the family caregivers (*n* = 11) classified themselves in the middle to upper annual family income level (> $46,000). Finances may impact the family’s ability to access private or additional supports and services. Further, the implications for practice are based on the specific patient contexts from the data. Additional contexts may arrive in other settings and thus may warrant further tailoring of information or services. Future research will test and validate the systems model in other health care settings.

## Conclusion

One size fits all discharge planning will not work. Rather, the caregiving role is adaptive to the context of care and dysfunctions in the stroke rehabilitation system. This role during the transition home from a rehabilitation facility supports continuity of care across health care settings. This study illustrates the family caregiver as a constant source of support and continuity for the stroke survivor during the transition from a rehabilitation facility to home. Due to the non-linearity, and dynamic nature of the system, the family caregivers were required to adapt to the changing environment, by shifting their role as the stroke survivor transitioned from a formal to an informal health care setting. As research has not yet empirically examined continuity of care using the complex adaptive systems lens, the findings enhance our understanding of family caregiving experience from a macro and micro perspective.

## Additional file

Additional file 1: Appendix A Coding Tree. The coding tree used to analyze the research data. (DOCX 98 kb)

## Abbreviation

CAS: Complex adaptive systems
