# Supplementary material for: Using a complex adaptive system lens to understand family caregiving experiences navigating the stroke rehabilitation system
Source: BMC Health Serv Res. 2016 Oct 1;16:538. doi: 10.1186/s12913-016-1795-6 (PMC5045641; doi:10.1186/s12913-016-1795-6)
Supplement: Additional file 1: — Appendix A Coding Tree. The coding tree used to analyze the research data. (DOCX 98 kb) [file 12913_2016_1795_MOESM1_ESM.docx]

**Appendix A:**

**Coding Tree**

**Informational Continuity**

Process of Information Exchange

(*The process by which information is provided, transferred or received*)

- Pushing information

*(The family caregivers provide the health professionals with information (i.e. the stroke survivor's health), or the health professionals who provide the family caregiver with information.)*

- Information retrieval (Pulling information)

*(The ability to get information from health professionals or other sources (i.e: electronic, paper).*

- Information Transfer

*(The transfer of information between health professionals, and/or health care settings.)*

Attributes of Information

(*Attributes might include the quality and/or reliability of the information, or type of information provided*)

Impacts

(*This might include an improved quality of care, ability to navigate the system, information overload, and access and coordination of services.*)

**Relational Continuity**

Patient/caregiver relationship

- Interaction – attributes

*(The might include discussion about the positive or negative nature of the relationship, and the amount of contact received or provided)*

Caregiver/Professional relationship

- Interaction – attributes

*(The might include discussion about the positive or negative nature of the relationship, and the amount of contact received or provided)*

Patient/Professional relationship

- Interaction – attributes

*(The might include discussion about the positive or negative nature of the relationship, and the amount of contact received or provided)*

**Management Continuity**

Process

- Follow up

(*Contact from health professional about the stroke survivors condition.* *This* *may be provided in the form of information, communication, services, assessment, check-in, or termination of services*)

- Timing

(*This might include the timing of decisions and information, the provision of resources, and/or the notice of transfer or discharge*)

- Problem-solving

*(Process of solving problems or figuring it out as you go; self-organization based on the message that 'you're on your own'; coming up with creative solutions)*

- Facility

(*Discussion surrounding a specific facility (i.e: Bruyère, St. Vincent’s), the facility’s rules / procedures for acute, rehab and outpatient services, and discussion about discharge, red tape, the availability of a case manager, and/or wait times for services at the facility*)

- Communication

(*This might include the channels used for information exchange or transfer, the transfer of information across settings.*)

Attributes of the case

- Professionals involved and relationships (between professionals / caregivers / patient)

*(This includes discussion about the health professionals involved in the case, and the formation of any relationships between the health professionals, patient or caregivers.)*

- Patient

*(This might include the stroke survivor’s mental, physical, or emotional health; functional capabilities; overall health)*

- Caregiver

*(This might include the family caregiver’s health literacy or knowledge of the health care system; their mental or physical health, and energy levels)*

- System

*(Discussion surrounding the health care system--this might include discussion about the availability of services; backlogs; caregivers taking up the slack when there are resource shortages)*

- Resources / social capital / social networks

*(A network of social connections—this might include social interactions with friends, family, and/or colleagues)*

Physical Environment

*(This refers to any mention of the physical environment; examples may include living in the country, changes that the families have to make to their homes to accommodate the care recipient, equipment needed in the house.)*

- Financial situation

(*Discussion about finances, and/or the impact the stroke has had on the family’s financial portfolio.)*

Impacts

*(This might include improved quality of care, improvements in the patient’s condition, stress or satisfaction, caregiver education etc.)*

**Caregiving role**

(*The family caregiver’s role as support provider at the rehabilitation facility, during the transition from the rehabilitation facility to home, and at home. Types of support might include instrumental, information or emotional support. The family caregiver’s inclusion or exclusion in the stroke survivors care; their role as first point of contact or system coordinator/navigator*)

**Recommendations**

(*Suggestions provided by the family caregiver in order to improve continuity of care in the stroke rehabilitation system, specifically during the transition home from a rehabilitation facility. Also, recommendations to ease the family caregiving role; recommendation for the facility etc.)*

**Transitions/Handoffs**

*(The stroke survivor’s movement between care settings or handoffs between health professionals.)*
